# Retinometer predicts visual outcome in Descemet membrane endothelial keratoplasty

**DOI:** 10.1007/s00417-022-05605-w

**Published:** 2022-02-26

**Authors:** Caroline Sophie Wald, Jan Darius Unterlauft, Matus Rehak, Christian Girbardt

**Affiliations:** 1 Department of Ophthalmology, University of Leipzig Medical Center, Liebigstrasse 10-14, 04103 Leipzig, Germany; 2 University Department of Ophthalmology, Inselspital, University of Bern, Bern, Switzerland; 3 Department of Ophthalmology, University Hospital of Gießen and Marburg, Gießen, Germany

**Keywords:** Retinometer, DMEK, Corneal transplantation, Interference visual acuity

## Abstract

**Purpose:**

To analyze the preoperative predictive value of retinometer visual acuity (VA) in eyes following Descemet membrane endothelial keratoplasty (DMEK).

**Methods:**

Patients undergoing DMEK between August 2011 and July 2020 were included. Preoperative interference visual acuity was assessed using Heine Lambda 100 Retinometer. Depending on the presence or absence of concomitant ocular disease, the Retinometer was evaluated for its ability to preoperatively predict best-corrected visual acuity (BCVA) six months after surgery using correlation, simple and multiple linear regression, contingency analyses, and receiver operating characteristic (ROC) analysis. Preoperative corneal backscatter was correlated with Retinometer prediction accuracy.

**Results:**

A total of 198 eyes were included in the analysis. There was a significant correlation between Retinometer VA and postoperative BCVA (*r* = 0.647, *P* < 0.001). Regardless of the presence or absence of concomitant ocular disease and the surgery procedure (DMEK & triple DMEK), Retinometer VA was the most significant predictor of postoperative BCVA (*P* < 0.001). ROC analysis revealed reliable diagnostic performance of the Retinometer (AUC = 0.829, *P* < 0.001). A Retinometer VA ≥ 0.5 accurately predicted a postoperative BCVA ≥ 0.5 in 91% of cases. No association was found between corneal backscatter and prediction accuracy (*P* = 0.566).

**Conclusions:**

Retinometer VA can be used for preoperative prediction of postoperative BCVA in DMEK and triple DMEK patients, independent of increased backscatter values and the presence or absence of concomitant ocular disease. By using this simple but effective tool, indication for DMEK can be facilitated and postoperative outcomes can be realistically predicted preoperatively.

**Supplementary Information:**

The online version contains supplementary material available at 10.1007/s00417-022-05605-w.


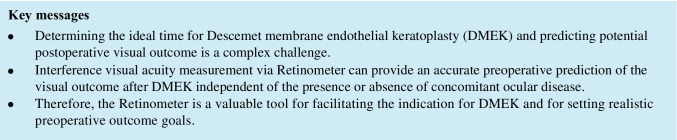


## Introduction

Since its introduction in 2006 [1], Descemet membrane endothelial keratoplasty (DMEK) has been established as a safe and effective treatment for patients suffering from corneal endothelial disease [2]. Benefits of DMEK include nearly full restoration of corneal anatomy, fast visual rehabilitation time, excellent visual outcome, and low complication rates [1–5]. Consequently, DMEK has gained worldwide popularity in recent years and is increasing at the highest rate of performed procedures compared to any other keratoplasty technique in the USA and in Germany [6, 7]. DMEK procedures are expected to reach the numbers of PK and DSAEK procedures in the next few years [7]. In Germany, DMEK is already the most frequently performed keratoplasty procedure [6].

When surgeons decide to perform DMEK, they and their patients are highly interested in the expected postoperative visual outcome, especially in the presence of concomitant ocular disease. Preoperative measurement of BCVA determines visual acuity status, which is influenced by concomitant ocular disease such as media opacities.

For this purpose, preoperative measurement of interference visual acuity (VA) can be used. The optical principle of interference VA is the projection of two coherent light beams into the eye, creating a striped pattern on the retina. This allows assessment of retinal resolving power in isolation [8, 9], largely independent of refractive errors and moderate opacities of refractive media [10, 11]. As a result, interference VA provides a preoperative estimation of expected postoperative VA. In clinical practice, a compact examination instrument called Retinometer can be used for the assessment of interference VA. This may help facilitate the indication for DMEK and avoid unnecessary surgery.

Previous studies have investigated the prognostic relevance of interference VA in cataract patients without [12–15] and with concomitant ocular disease [16–22] and in patients suffering from epiretinal gliosis [23–25]. Analyses in patients undergoing penetrating keratoplasty were performed using laser interferometer [26–28] and potential acuity meter [28, 29]. So far, no interference VA measurements in DMEK patients have been published.

The extent of corneal decompensation as detected by corneal density measurements may interact with interference VA, as increased corneal backscatter values have been found in patients with Fuchs endothelial dystrophy (FED) at all stages of disease [30].

The purpose of this study was to evaluate the accuracy of interference VA in patients undergoing DMEK and to identify cutoff values that preoperatively predict DMEK outcome. Furthermore, the effect of corneal decompensation and presence of concomitant ocular disease on the accuracy of interference VA measurement were investigated.

## Material and methods

### Study design

Retrospective interventional case series.

### Study participants

Data from patients receiving DMEK at the Department of Ophthalmology, University of Leipzig Medical Center, Germany, between August 2011 and July 2020 was reviewed. Retrospective data was collected until December 2018, with Retinometer VA documented in 212 cases. Since January 2019, the study was continued prospectively by systematically measuring interference VA in all DMEK patients (*n* = 69).

DMEK was performed by two experienced surgeons (CG, JDU) according to the technique published by Melles [1] and modified by Cursiefen [31]. In patients with concomitant cataract, phacoemulsification and posterior chamber lens implantation (triple DMEK) were performed within the same session.

### Retinal visual acuity

Interference VA measurements were performed using Heine® Retinometer (Lambda 100® Retinometer, Heine Optotechnik GmbH & Co. KG, Gilching, Germany), a hand-held device based on the principle of light interference. The device shows a spot of light projecting a circular interference pattern of alternating black and red stripes onto the patient’s retina [10, 11, 19]. Both fineness and stripe pattern direction (horizontal, diagonal, vertical) can be varied to determine the finest stripe width at which pattern orientation is still correctly recognized by the patient. The Retinometer provides a range of seven distinct values within a VA range of 0.07 to 0.80. Starting with the broadest pattern, a random stripe orientation was chosen. If recognized correctly by the patient, a finer stripe pattern and a new orientation were set. The value of the finest recognizable stripe pattern was recorded as Retinometer VA. According to the manufacturer’s recommendations, measurements were performed in reduced ambient lighting and with dilated pupils. Although a measurement with optical correction is not necessary due to the independence of refractive errors [10, 11], we performed the measurements with best-spectacle correction to guarantee generalizability of the results.

### Data collection

Preoperative central corneal thickness (CCT) and corneal backscatter in grey scale units (GSU) of the central annular zone at 0–2 mm from anterior corneal layer [32] were measured using Scheimpflug-based Oculus® Pentacam® (Oculus GmbH, Wetzlar, Germany). Concomitant ocular disease and postoperative BCVA six months after DMEK were assessed.

Exclusion criteria were intraoperative and postoperative complications that, in the surgeon’s judgment, affected postoperative VA. Furthermore, subjects with inadequate cooperation or comprehension problems during Retinometer measurement leading to unreliable interpretation of data were excluded.

### Classification of ocular comorbidities

Concomitant ocular disease was classified into five categories: (1) anterior segment pathology relevant to VA, (2) central fundus and vitreous body pathology relevant to VA, (3) glaucoma and other optic nerve defects, (4) amblyopia and non-organic visual loss, and (5) ocular conditions not relevant to VA. Patients with nonorganic vision loss were defined as deterioration of vision despite normal ophthalmologic examination including electrophysiologic testing. Patients were classified accordingly, with patients without concomitant ocular disease assigned to category 5. Multiple assignments within the first four categories per patient were possible. Allocations of each ocular pathology to the five categories are presented in Online Resource Table 1.

### Classification of estimation accuracy

In order to analyze Retinometer prediction accuracy, eyes were categorized as in previous Retinometer studies [12]: underestimation (Retinometer VA < BCVA), overestimation (Retinometer VA > BCVA), and accurate estimation (Retinometer VA ≙ BCVA).

As described by Rassow et al. [33], a postoperative BCVA range for which Retinometer prediction was considered correct was defined for each preoperative Retinometer VA value. Eyes with postoperative BCVA above one level of preoperative Retinometer VA were categorized as underestimation, whereas eyes with postoperative BCVA below one level of preoperative Retinometer VA were categorized as overestimation. If the BCVA was accurate or within ± 1 lines of preoperative Retinometer measurement, this was interpreted as an accurate estimation.

### Statistical analysis

Data was analyzed using SPSS® (version 23 for Windows; SPSS Inc., Chicago, IL, USA). The scatterplot was created with R (version 4.1.0 for Windows; R Core Team, R Foundation For Statistical Computing, Vienna, Austria). If data from a patient’s right and left eyes were eligible, one eye was randomly selected according to the recommendation of Armstrong et al. [34], in order to exclude statistical bias due to possible correlation between the left and right eye of a subject. Preoperative Retinometer VA and postoperative BCVA were transformed into logarithmically graded ranks as shown in Online Resource Table 2 (modified according to Schulze et al. [20]). Transformation into ranks was performed to include low VA (hand movement = 1, finger counting = 2) and to prevent bias in the statistical analysis due to the varying distances between the values.

Retinometer prediction accuracy was defined as BCVA minus Retinometer VA. The proportion of variance explained (PVE) in postoperative BCVA rank by Retinometer VA rank and concomitant ocular disease categories was computed following Shim et al. [35].

We performed descriptive analysis and Fisher’s exact test for analysis of categorical variables. Correlations were performed using Pearson coefficient r. Analogous to the interpretation in Schaub et al. [36] correlation was interpreted as strong (*r* ± 0.70), moderate (*r* ± 0.50), or weak (*r* ± 0.30), depending on which value *r* was closest to. Simple and multiple linear regressions were performed to assess the predictive value of the independent variables Retinometer VA and individual concomitant ocular disease. Diagnostic performance was evaluated by using receiver operating characteristic (ROC) analysis to calculate the area under the curve (AUC). The higher the AUC, the more accurate the test. Sensitivity, specificity, positive (PPV), and negative predictive value (NPV) were calculated. Sensitivity indicates the percentage of patients with BCVA ≥ 0.5 for whom the Retinometer indicated VA ≥ 0.5. Specificity indicates the percentage of patients with BCVA < 0.5 for whom the Retinometer indicated VA < 0.5. The PPV represents the patient percentage with a Retinometer VA ≥ 0.5 that had a BCVA ≥ 0.5. The NPV states the percentage of patients with a Retinometer VA < 0.5 who had a BCVA < 0.5.

The level of significance was defined as *P* < 0.05.

## Results

A total of 281 eyes were examined. Afterwards, one eye was randomly selected in subjects for whom data were available from both eyes. There was a moderate negative correlation (*n* = 206, *r* = -0.554, *P* < 0.001) between corneal thickness and Retinometer VA rank. Outliers within the normal distribution of corneal thickness were identified in corneas more than twice as thick than the average of 550 µm. To avoid statistical bias, only eyes with corneal thickness values ≤ 1100 µm (*n* = 198) were included for further analysis.

Descriptive analysis and clinical outcomes of the study cohort are presented in Table 1.

**Table 1:** Clinical baseline data of the study cohort (*n* = 198)

		Mean ± SD	
Age (years)		72.4 ± 7.8	
Preoperative retinometer VA		0.5 ± 0.2	
Postoperative BCVA		0.6 ± 0.2	
Preoperative corneal thickness (µm)		661.3 ± 89.8	
Preoperative corneal backscatter (GSU, *n* = 39)		47.6 ± 19.9	
		n	(%)
Gender	Female	134	(68)
	Male	64	(32)
Surgery	Pseudophakic DMEK	119	(60.1)
	Triple DMEK	79	(39.9)
Indication	Fuchs endothelial dystrophy	178	(89.9)
	Pseudophakic bullous keratopathy	14	(7.1)
	Re-DMEK	6	(3.0)
Presence of ocular comorbidities		109	(55.1)
	Anterior segment pathology relevant to VA	41	(20.7)
	Central fundus and vitreous body pathology relevant to VA	56	(28.3)
	Glaucoma and other optic nerve defects	36	(18.2)
	Amblyopia and non-organic VA loss	8	(4)
Ocular conditions not relevant to VA		89	(44.9)

**Fig. 1:**
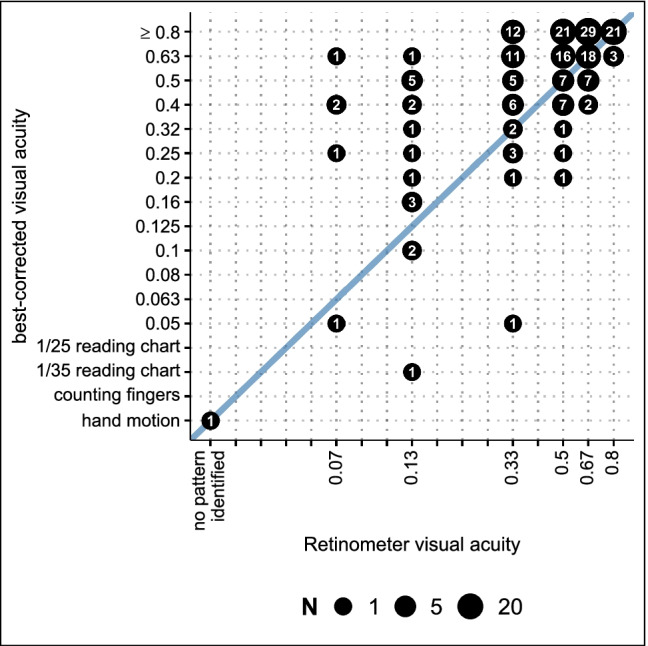
Correlation between preoperative Retinometer VA ranks and BCVA ranks (Pearson’s correlation coefficient *r* = 0.647, *P* < 0.001)

There was a moderate positive correlation between Retinometer VA rank and BCVA rank (*n* = 198, *r* = 0.647, *P* < 0.001). Retinometer VA rank correlated significantly with BCVA rank both in DMEK alone and triple DMEK (DMEK, *n* = 119, *r* = 0.668, *P* < 0.001; triple DMEK, *n* = 79, *r* = 0.321, *P* < 0.001). Comparison of postoperative BCVA rank and preoperative Retinometer VA rank is displayed in Fig. 1.

Within each category of existing concomitant ocular disease, there was a strong correlation between Retinometer VA rank and BCVA rank: (1) anterior segment pathology relevant to VA (*n* = 41, *r* = 0.788, *P* < 0.001) and (4) amblyopia and non-organic visual loss (*n* = 8, *r* = 0.769, *P* = 0.05). Within the category (2) central fundus and vitreous body pathology relevant to VA (*n* = 56, *r* = 0.507, *P* < 0.001) and (3) glaucoma & other optic nerve defects (*n* = 36, *r* = 0.656, *P* < 0.001), there was a moderate correlation.

Overall, the Retinometer demonstrated accurate estimation accuracy and was not affected by the presence of different concomitant ocular pathologies (Table 2).

**Table 2:** Retinometer prediction accuracy depending on concomitant ocular disease

	Underestimation		Accurate estimation		Overestimation		Total
	*n*	(%)	*n*	(%)	*n*	(%)	*n*
Anterior segment pathology relevant to VA	12	29.3%	29	70.7%	0	0.0%	53
Central fundus and vitreous body pathology relevant to VA	15	26.8%	39	69.6%	2	3.6%	73
Glaucoma and other optic nerve defects	10	27.8%	26	72.2%	0	0.0%	47
Amblyopia and non-organic	1	12.5%	6	75%	1	12.5%	14

There was no significant correlation between prediction accuracy and corneal backscatter (*n* = 39, *r* = -0.095, *P* = 0.566).

Simple linear regression models showed that Retinometer VA rank was a significant predictor of BCVA rank, independent of the presence (*n* = 109, B coefficient = 0.596, β coefficient = 0.617, *P* < 0.001, 95% confidence interval [CI] 0.45 to 0.74, adjusted *R*^2^ = 0.375) or absence of concomitant ocular disease (*n* = 89, B coefficient = 0.296, β coefficient = 0.505, *P* < 0.001, 95% CI 0.18 to 0.40, adjusted *R*^2^ = 0.247).

A multiple linear regression analysis incorporating Retinometer VA rank and the four categories of existing concomitant ocular disease as potential interacting predictors of postoperative BCVA rank (*n* = 198, *P* < 0.001, adjusted *R*^2^ = 0.529) showed all variables as significant predictors of postoperative BCVA rank. Among all variables, the proportion of variance in the rank of BCVA was most significantly explained by the rank of Retinometer VA (*n* = 198, B coefficient = 0.464, β-coefficient = 0.507, *P* < 0.001, 95% CI 0.37 to 0.56, PVE = 0.313).

**Fig. 2:**
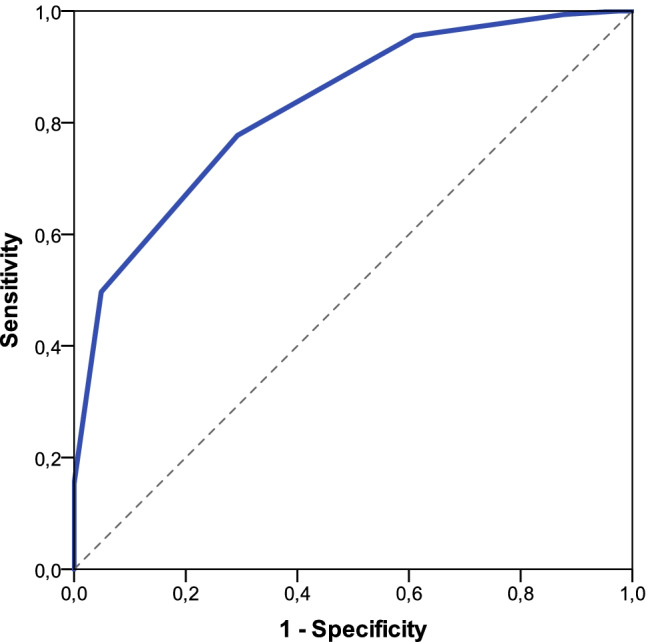
Receiver operating characteristic analysis assessing the sensitivity and specificity of the Retinometer for discriminating between postoperative BCVA ≥ 0.5 and < 0.5

The ROC curve assessing diagnostic performance for discriminating between postoperative BCVA ≥ 0.5 and < 0.5, respectively, had an AUC of 0.829 (95% CI 0.76 to 0.90 *P* < 0.001) and is displayed in Fig. 2. Based on this analysis, a Retinometer VA rank cutoff ≥ 14, corresponding approximately to a Retinometer VA ≥ 0.5, had a sensitivity of 77.7% and a specificity of 70.7% for predicting postoperative BCVA ≥ 0.5.

The significant association between Retinometer VA ≥ 0.5 and BCVA ≥ 0.5 was confirmed using Fisher’s exact test (*n* = 198, *P* < 0.001), with a sensitivity of 77.7% and a specificity of 70.7%. If the Retinometer predicted VA ≥ 0.5, BCVA was ≥ 0.5 in 91% of cases (PPV). However, in 45.3% of patients with a Retinometer VA < 0.5, the postoperative BCVA was < 0.5 (NPV). In only 8.96% of cases, postoperative BCVA was < 0.5 despite a Retinometer prediction of VA ≥ 0.5.

There was a significant association between Retinometer VA ≥ 0.5 and postoperative BCVA ≥ 0.5 within the comorbidity categories anterior segment pathology relevant to VA (*P* = 0.017), VA relevant changes of the central fundus and vitreous body (*P* < 0.001), and glaucoma and other optic nerve defects (*P* < 0.001), but not within the category amblyopia and non-organic VA loss (*P* = 1.00).

## Discussion

This study is the first to systematically analyze the prognostic relevance of preoperative interference VA measurements in patients undergoing DMEK. For both the surgeon and the patient, a preoperative parameter should be determined that can facilitate decision-making for performing DMEK and provide prognostically significant information. In FED patients, several parameters have already been identified to facilitate decision-making and assist in predicting postoperative outcome after DMEK: preoperative BCVA [37], corneal backscatter [30, 32, 36] and other densitometry parameters, preoperative corneal thickness [32, 38], the course of visual restitution of the first eye [38], and the graft’s baseline endothelial cell density [38]. Nevertheless, none of the parameters examined in studies so far could provide an accurate preoperative prediction of the visual outcome after DMEK.

Patients with concomitant ocular diseases were mostly excluded in previous studies. To better transfer the results to clinical practice, patients with concomitant ocular disease were included in this study.

The Retinometer can be used to predict the expected postoperative BCVA by measuring the resolving power of the retina in isolation [8, 9], largely independent of refractive errors and of moderate opacities of the refractive media [10, 11].

In patients receiving penetrating keratoplasty, interference VA has been identified as a valuable tool for clinical decision-making for preoperative assessment of postoperative BCVA, even in presence of additional concomitant ocular disease [26–28]. In general, good preoperative interferometric VA was identified as being predictive for good postoperative VA, whereas poor interferometric VA in patients having opacified media did not predict good postoperative VA [27]. In these studies, the Pearson correlation coefficient was not determined. According to Enoch et al. [27], the Retinometer underestimated or accurately estimated postoperative BCVA in 55.6% of cases, compared to 70% of cases as reported by Gstalder et al. [26] and 69.9% as reported by Steinert et al. [28].

The Retinometer showed a moderate tendency to underestimate VA in previous cataract studies [10, 12] and a tendency to overestimate VA in the presence of amblyopia [10, 23, 26, 33] or maculopathy [23].

In this study, the Retinometer revealed an accurate estimation tendency in the majority of cases. Nevertheless, the rate of underestimation was higher than the rate of overestimation. Furthermore, Retinometer VA was shown to be a significant predictor of BCVA after DMEK, regardless of the presence or absence of concomitant ocular disease. Because backscatter did not affect prediction accuracy, the Retinometer can be used reliably in patients with corneal decompensation, as occurs during the course of corneal endothelial diseases [30]. There was a significant correlation between Retinometer VA rank and BCVA rank in both DMEK alone and triple DMEK, demonstrating the Retinometer’s ability for preoperative VA prediction in both contexts.

Additionally, the Retinometer was capable of significantly discriminating between postoperative BCVA ≥ and < 0.5 within each category of existing ocular comorbidity, except for patients suffering from amblyopia and non-organic VA loss. Furthermore, it had a very high PPV (91%). If Retinometer VA is good, the indication for DMEK can be given without hesitation and with prospect of positive outcome. In only 45.3% of patients with Retinometer VA < 0.5, postoperative BCVA was < 0.5 (NPV). This indicates that if Retinometer VA is poor, the patient may still benefit from surgery. Accordingly, surgery should not be rejected purely based on poor Retinometer VA. This observation supports the assumption that the Retinometer may tend to moderately underestimate postoperative VA, meaning that in clinical practice, postoperative BCVA is mostly as good as or even better than the predicted Retinometer VA.

Because predicted VA was higher than the actual postoperative BCVA in only 8.96% of the cases, there is little concern about a potentially disappointing overestimation of postoperative VA through the Retinometer.

A limitation of this study is that Retinometer measurements were performed in the retrospective cases only if the indication for DMEK was controversial. Prospective data of all subsequent DMEK patients collected was able to mitigate this selection bias. There might be several reasons why the correlation coefficient of 0.647 has only moderate strength. Both measurement methods are subjective and rely on the correct collaboration with the patient. There was no averaging of the values by repeated measurements. Furthermore, Retinometer is not capable of measuring precisely the same metric values as in BCVA measurement, but only certain levels. Categorization was used to reduce this bias.

In addition, the numbers of cases within each comorbidity category, especially in amblyopia and non-organic visual loss, and the number of corneal backscatter values were relatively low. Due to this fact, a prospective study with higher case numbers would be desirable. Another limitation is the potentially low generalizability of the study results due to the study cohort predominantly consisting of patients with Fuchs endothelial dystrophy.

In conclusion, this study demonstrates the prognostic relevance of preoperative Retinometer VA for postoperative BCVA six months after DMEK. The Retinometer can be used even in the presence of concomitant ocular disease. Therefore, the Retinometer is a valuable tool for facilitating the indication for DMEK, for avoiding unnecessary surgery, and for setting realistic preoperative outcome goals.

## Supplementary Information

Below is the link to the electronic supplementary material.

Supplementary file1 (DOCX 18 KB)
