# Supplementary material for: Retinometer predicts visual outcome in Descemet membrane endothelial keratoplasty
Source: Graefes Arch Clin Exp Ophthalmol. 2022 Feb 26;260(7):2283–90. doi: 10.1007/s00417-022-05605-w (PMC9203399; doi:10.1007/s00417-022-05605-w)
Supplement: Supplementary file 1 — Supplementary file1 (DOCX 18 KB) [file 417_2022_5605_MOESM1_ESM.docx]

**Graefe's Archive for Clinical and Experimental Ophthalmology**

# **Retinometer Predicts Visual Outcome in Descemet Membrane Endothelial Keratoplasty**

Caroline Sophie Wald^1^, Jan Darius Unterlauft^2^, Matus Rehak^3^, Christian Girbardt^1^

^1^*Department of Ophthalmology, University of Leipzig Medical Center, Leipzig, Germany*

^2^*University Department of Ophthalmology, Inselspital, University of Bern, Bern, Switzerland*

^3^Department *of* Ophthalmology, University Hospital *of* Gießen *and Marburg, Gießen, Germany*

Corresponding Author: Dr. Christian Girbardt, Email: christian.girbardt@medizin.uni-leipzig.de

**Online Resource Table 1** Classification of concomitant ocular disease

|  | **Category** | **Concomitant ocular disease** | **Number** |
| --- | --- | --- | --- |
| **Anterior segment pathology relevant to VA** | | Cataract, aftercataract, corneal scars, iridoplegia, Axenfeld-Rieger syndrome, s/p perforating injury, s/p perforating corneal ulcer, s/p keratitis, corneal dystrophies: epithelial basement membrane dystrophy, lattice corneal dystrophy | 41 |
| **Central fundus & vitreous body pathology relevant to VA** | | Age-related macular degeneration (exudative/non-exudative), myopic maculopathy, diabetic retinopathy with macular edema, epiretinal gliosis, macular lamellar hole, cystoid macular edema, perifoveolar telangiectasia, pseudovitelliform macular degeneration, pattern dystrophy, asteroid hyalosis, central vitreous opacities, s/p posterior uveitis | 56 |
| **Glaucoma & other optic nerve defects** | | Primary chronic open angle glaucoma, chronic angle closure glaucoma, pseudoexfoliation glaucoma, secondary glaucoma, optic atrophy, anterior ischemic optic neuropathy | 36 |
| **Amblyopia & non-organic visual loss** | | Amblyopia, microtropia, visual loss without morphological correlate | 8 |
| **Ocular conditions not relevant to VA** | | Eyelid cyst, lacrimal duct stenosis, s/p iritis, s/p scleritis, nystagmus, sixth nerve palsy, secondary exotropia, s/p eye contusion, dry eye disease, choroidal nevus, non-proliferative diabetic retinopathy without macular edema, retinal artery branch occlusion, s/p retinal detachment without macular involvement, aphakia, Salzmann degeneration, postoperative cystoid macular edema (completely resorbed within study period), papillary hamartoma | 89 |

**Online Ressource Table 2** Transformation of Retinometer VA and postoperative BCVA into ranks

| **Retinometer VA** | **Retinometer VA rank** | **BCVA** | **BCVA rank** |
| --- | --- | --- | --- |
| 0.8 | 17 | ≥ 0.8 | 17 |
| 0.67 | 16 | 0.63 | 16 |
| 0.5 | 15 | 0.5 | 15 |
| 0.33 | 13 | 0.4 | 14 |
| 0.13 | 9 | 0.32 | 13 |
| 0.07 | 6 | 0.25 | 12 |
| no pattern identified | 1 | 0.2 | 11 |
|  |  | 0.16 | 10 |
|  |  | 0.125 | 9 |
|  |  | 0.1 | 8 |
|  |  | 0.08 | 7 |
|  |  | 0.063 | 6 |
|  |  | 0.05 | 5 |
|  |  | 1/25 reading chart | 4 |
|  |  | 1/35 reading chart | 3 |
|  |  | counting fingers | 2 |
|  |  | hand motion | 1 |
